# Anti-Photoaging Effect of Jeju Putgyul (Unripe Citrus) Extracts on Human Dermal Fibroblasts and Ultraviolet B-induced Hairless Mouse Skin

**DOI:** 10.3390/ijms18102052

**Published:** 2017-09-25

**Authors:** Seung-Hyun Choi, Sun-Il Choi, Tae-Dong Jung, Bong-Yeon Cho, Jin-Ha Lee, Seung-Hyung Kim, Seon-A Yoon, Young-Min Ham, Weon-Jong Yoon, Ju-Hyun Cho, Ok-Hawn Lee

**Affiliations:** 1 Department of Food Science and Biotechnology, Kangwon National University, Chuncheon 24341, Korea; zzaoszz@naver.com (S.-H.C.); docgotack89@hanmail.net (S.-I.C.); lgtjtd@naver.com (T.-D.J.); bongyeon.cho92@gmail.com (B.-Y.C.); tre98@hanmail.net (J.-H.L.); 2 Institute of Traditional Medicine and Bioscience, Daejeon University, Daejeon 34520, Korea; sksh518@dju.kr; 3 Jeju Biodiversity Research Institute (JBRI), Jeju Technopark (JTP), Jeju 63608, Korea; yoonsa33@jejutp.or.kr (S.-A.Y.); hijel@jejutp.or.kr (Y.-M.H.); yyjkl@jejutp.or.kr (W.-J.Y.); 4 Hurum Central Research Institute, Seogwipo 63608, Korea

**Keywords:** *Citrus unshiu* S.Marcov, human dermal fibroblasts, hairless mice, anti-photoaging, matrix metalloproteinase

## Abstract

Ultraviolet (UV) radiation stimulates the expression of matrix metalloproteinases (MMPs) and inflammatory cytokines. These signaling pathways participate in the degradation of the extracellular matrix and induce inflammatory responses that lead to photoaging. This study evaluated the antioxidant activity and the effect on MMPs and procollagen of putgyul extract in vitro. The anti-photoaging activity of putgyul extracts was estimated in vivo using hairless mice (HR-1). The putgyul extracts reduced MMP-1 production and increased the content of procollagen type I carboxy-terminal peptide in human dermal fibroblasts. Ultravilot-B (UVB)-induced expression of inflammatory cytokines and MMPs was detected in mice, and putgyul extracts suppressed the expression. These results suggest that putgyul extract inhibits photoaging by inhibiting the expression of MMPs that degrade collagen and inhibiting cytokines that induce inflammatory responses. The mouse model also demonstrated that oral administration of putgyul extracts decreased wrinkle depth, epidermal thickness, collagen degradation, and trans-epidermal water loss, and increased β-glucosidase activity on UVB exposed skin. Putgyul extract protects against UVB-induced damage of skin and could be valuable in the prevention of photoaging.

## 1. Introduction

The skin is the largest organ of the human body. It comprises various layers, including the epidermis and dermis. These layers form a protective barrier from environmental stresses, such as heat, infection, oxidative activity, and water loss [1,2,3]. Skin aging occurs due to two biochemical processes. Intrinsic aging refers the progressive aging that occurs with time with continued exposure to ultraviolet (UV) light [4]. UV light can be classified according to wavelength as ultraviolet-C (UVC) (200–280 nm), ultraviolet-B (UVB) (280–320 nm), and ultraviolet-A (UVA) (320–400 nm). While UVC emitted by the sun does not pass through the ozone (O_3_) layers of the Earth’s atmosphere, UVA and UVB can penetrate through the atmosphere to the surface of the earth [5,6]. Among these, UVB mainly induces changes in the epidermis. Photodamaged skin is characterized by the development of brown spots, irregular pigmentation, reduced elasticity, drying, and formation of wrinkles [7,8,9].

UV-induced skin damage involves a variety of mechanisms, such as collagenase and inflammatory cytokine activities [10]. UV exposure of skin promotes the production of nuclear factor κB (NF-κB) and activator protein-1 (AP-1). Both stimulate inflammatory cytokines, such as interleukin (IL)-1β (IL-1β), IL-6 and tumor necrosis factor-α (TNF-α). These inflammatory cytokines stimulate the accumulation of reactive oxygen species (ROS) that damage skin tissues and cells, and increase the expression of matrix metalloproteinases (MMPs) that destroy the structure and function of the extracellular matrix (ECM) by degrading collagen. UV exposure also stimulates the production of cyclooxygenase-2 (COX-2), which aggravates skin inflammation by catalyzing the synthesis of prostaglandin E_2_ (PGE_2_) and the overexpression of inducible nitric oxide synthase (iNOS), which causes skin inflammation [11,12,13].

This study examined the effects of putgyul in ameliorating the detrimental photoaging effects of UV on skin. Putgyul is a term used in Korea to describe *Citrus unshiu* S.Marcov orange. Citrus fruits such as oranges contain organic acids, vitamins, minerals, citric acid, ascorbic acid and flavonoids [14,15]. Unripe citrus fruit is more enriched than ripe fruit in dietary fiber, polyphenol, and flavonoids such as hesperidin, and naringin [16]. 

Most prior studies have examined ripe citrus. The influence of putgyul on photoaging is unknown. This study investigated the anti-photoaging effects of putgyul extracts on human dermal fibroblasts (HDFs) and UVB-induced damage of the skin of a hairless mouse model.

## 2. Results

### 2.1. Antioxidant Activity of Putgyul Extracts

A 2,2-diphenyl-1-picrylhydrazyl (DPPH) radical scavenging assay was used to measure the antioxidant activity of putgyul extracts. Scavenging activity is expressed as the half maximal inhibitory concentration (IC_50_), which is the concentration required to scavenge 50% of the DPPH radicals. The DPPH radical scavenging activity of putgyul extracts, calculated from regression lines using five different concentrations in triplicate experiments, was 788.46 ± 0.45 µg/mL.

### 2.2. Putgyul Extracts Improve Intracellular Collagen and Matrix Metalloproteinase (MMP)-1 in Human Dermal Fibroblasts (HDFs)

**Figure 1:**
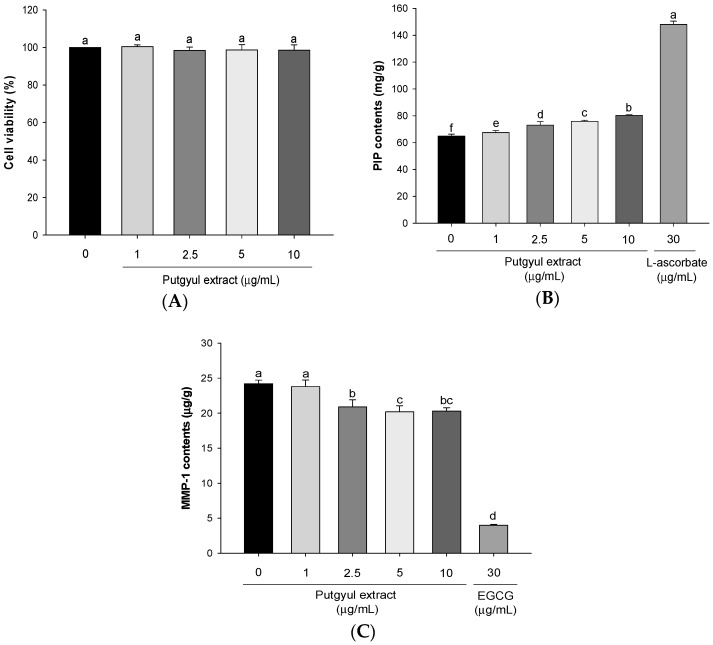
Effects of putgyul extracts on collagen contents and matrix metalloproteinase (MMP)-1 in human dermal fibroblasts (HDFs). (**A**) Cell viability of putgyul extracts in HDF; (**B**) PIP contents in HDF after treatment with putgyul extracts; (**C**) MMP-1 contents in HDF after treatment of unripe citrus extracts. ^a–f^ All values are presented as mean ± standard deviation. Bars with different letters indicate statistically significant differences among the groups at *p* < 0.05 by one-way ANOVA. EGCG, epigallocatechin gallate.

MMP-1 (also termed collagenase) degrades ECM components like type 1 collagen during the aging process [17]. HDFs were used for an in vitro evaluation of changes in MMP and collagen that affect skin wrinkle formation. The effects of putgyul extracts on intracellular collagen and MMP-1 were determined at extract concentrations that did not affect cell viability (Figure 1A). The content of the intracellular collagen, procollagen type I carboxy-terminal peptide (PIP), was significantly increased depending on the concentration of putgyul extracts (Figure 1B). Intracellular collagenase (MMP-1) activity and MMP-1 production were significantly inhibited by the putgyul extracts in a dose-dependent manner (Figure 1C). These results suggest that putgyul extracts suppress collagen degradation through the inhibition of MMP.

### 2.3. Putgyul Extract Inhibits Inflammatory Cytokines in UVB-Induced Hairless Mice

**Figure 2:**
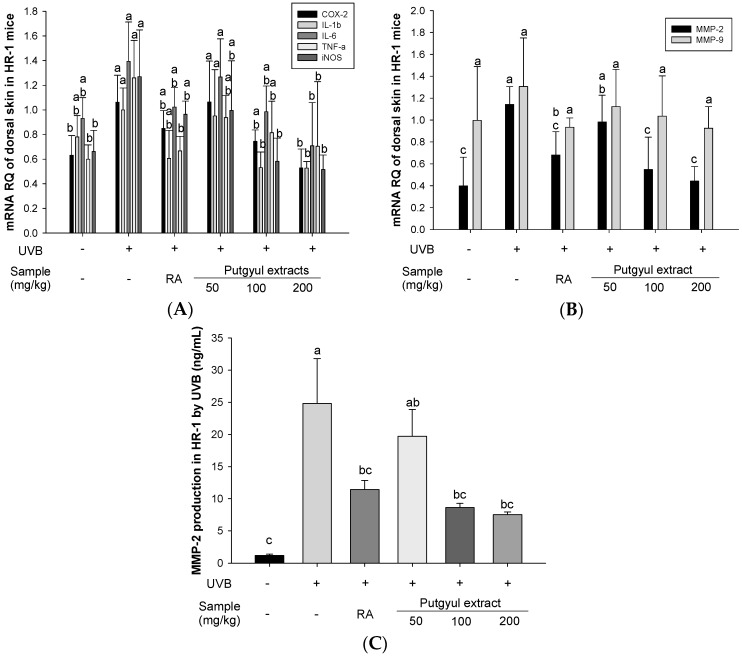
Effects of putgyul extracts on expression of cytokines and protein in ultraviolet-B (UVB)-induced hairless mice. (**A**) Expression levels of the inflammatory cytokines; (**B**) mRNA expression of MMPs; (**C**) MMP-2 protein expression. ^a^^–^^c^ All values are presented as mean ± standard deviation. Bars with different letters indicate statistically significant differences among the groups at *p* < 0.05 by one-way ANOVA. mRNA results are expressed as the relative quantity.

UVB-induced skin damage is associated with the production of inflammatory cytokines [17]. To determine the effects of putgyul extracts on inflammatory cytokines, we investigated the effects of putgyul extracts on mRNA expression of COX-2, IL-1β, IL-6, TNF-α, and iNOS. The expression of these inflammatory cytokines was increased by UVB irradiation. However, putgyul extract treatment decreased the expression of all five cytokines in a concentration-dependent manner (Figure 2A). The observations suggest that putgyul extracts inhibit the inflammatory response.

### 2.4. Putgyul Extract Inhibits mRNA and Protein Expression of MMPs In Vivo

The MMP-2 and MMP-9 gelatinases have been associated with collagen degradation [18]. To confirm the effect of putgyul extracts on MMPs, MMP-2 and MMP-9 mRNA expression was measured (Figure 2B). UV-induced mice displayed increased expression of MMP-2 and MMP-9 mRNA. Treatment with putgyul extracts suppressed the mRNA expression relative to UV-vehicle group, with the difference being significant for MMP-2. The protein expression of MMP-2 was measured (Figure 2C) to observe the changes in wrinkle formation caused by skin collagen degradation by UVB photoaging. MMP-2 production was increased in the UVB-treated group compared to the normal control group. However, MMP-2 production was significantly decreased in a dose-dependent manner when putgyul extracts were administered. The findings indicated that putgyul extracts block collagen degradation by inhibiting MMP activity.

### 2.5. Putgyul Extract Inhibits UVB-Induced Photoaging In Vivo

**Figure 3:**
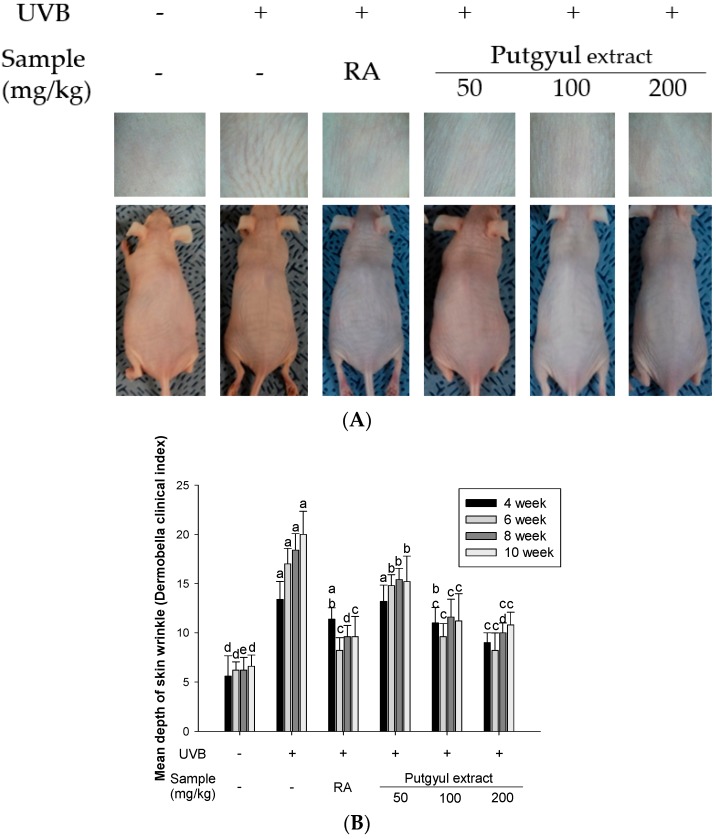
Effect of putgyul extracts on UVB-induced wrinkle formation in hairless mice. (**A**) Features of dorsal skin of hairless mice exposed to UVB; (**B**) Mean of skin wrinkle depth. ^a–d^ All values are presented as means ± SD. Bars with different letters indicate statistically significant differences among the groups at *p* < 0.05 by one-way ANOVA.

The inhibitory effect of retinoic acid and putgyul extracts on wrinkle formation was assessed in UVB-induced hairless mice. The mice were exposed to UVB for 10 weeks. The effect on skin morphology in terms of dorsal wrinkles and skin wrinkle depth was observed (Figure 3). Skin wrinkle formation and wrinkle depth were increased in the UVB-induced group compared to the normal group. UVB-induced wrinkle depth was reduced in mice treated with retinoic acid and putgyul extracts. Wrinkle depth development was suppressed by the extracts in a dose-dependent manner. In particular, at high concentrations, there was no significant difference from the positive control retinoic acid group.

### 2.6. Putgyul Extract Inhibits Increased Epidermal Thickness and Collagen Degradation by UVB-Induced Photoaging In Vivo

**Figure 4:**
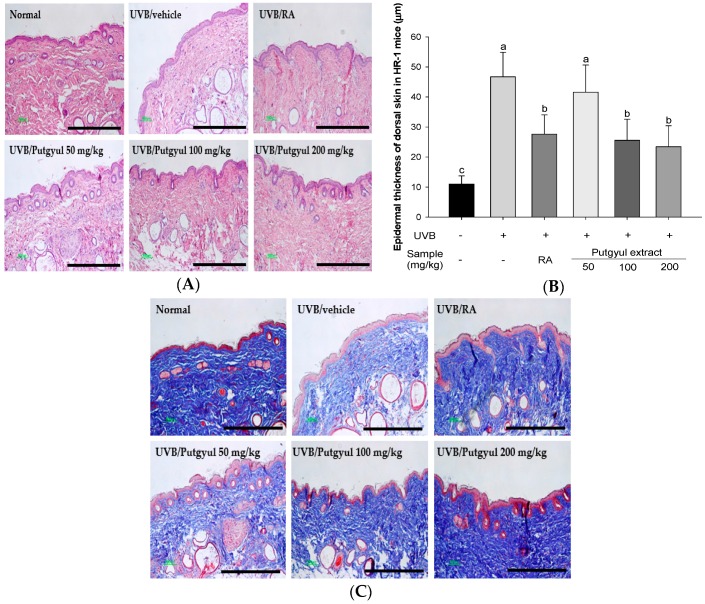
Histological analyses of hematoxylin and eosin (H&E) and collagen degradation in hairless mouse skin. (**A**) H&E stained sections; (**B**) Epidermal thickness; (**C**) Collagen fibers were stained using Masson’s trichrome stain. Scale bar is 400 µm. ^a^^–^^c^ All values are presented as mean ± standard deviation. Bars with different letters indicate statistically significant differences among the groups at *p* < 0.05 by one-way ANOVA.

Photoaging of skin involves increased epidermal thickness and collagen degradation [19]. Histological analysis of dorsal skin of the hairless mice was done to evaluate the anti-photoaging effects of putgyul extracts on epidermal thickness and collagen degradation. H&E staining revealed significantly increased epidermal thickness in UVB irradiated mice. Mice treated with putgyul extracts displayed a dose-dependent decreased skin thickness (Figure 4). Masson’s trichrome stain was used to identify collagen fibers, which stain blue. Staining of the collagen fibers was significantly decreased in the UVB control group compared to the normal group, whereas staining of the fibers was increased in tissue from mice treated with the putgyul extracts compared to the UVB-vehicle group (Figure 4C). These results suggest that putgyul extract inhibits collagen degradation and modifies the effect of UVB irradiation on epidermal thickness.

### 2.7. Putgyul Extract Improves Transepidermal Water Loss (TEWL) and β-Glucosidase Activity In Vivo

**Figure 5:**
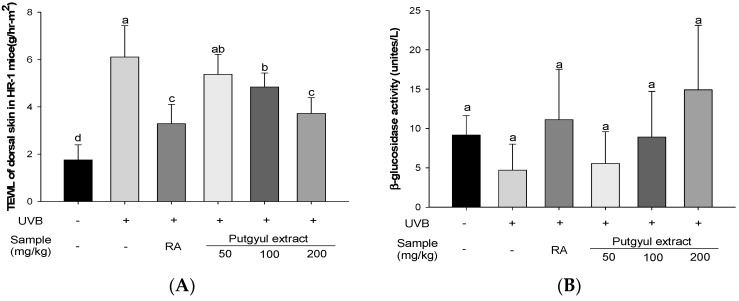
Moisturizing effect of putgyul extracts in UVB-induced hairless mice. (**A**) Transepidermal water loss (TEWL) of dorsal skin; (**B**) β-glucosidase activity. ^a–c^ All values are presented as mean ± standard deviation. Bars with different letters indicate statistically significant differences among the groups at *p* < 0.05 by one-way ANOVA.

Hairless mice were irradiated with UVB to induce wrinkles. After 10 weeks, the skin moisture content and β-glucosidase activity were evaluated. Skin moisture content was determined to evaluate the barrier function of UV-induced skin of mice by TEWL. TEWL of the UVB-vehicle group was increased compared to the normal group. TEWL of mice treated with putgyul extracts and retinoic acid was significantly lower than the UVB-vehicle group. In particular, UVB-irradiated mice treated with putgyul extracts displayed TEWL that was decreased by the extracts in a dose-dependent manner (Figure 5A). β-glucosidase is an enzyme that functions in the regeneration of ceramide from glycosylceramide and acylglycosylceramide [20]. The β-glucosidase activity in the UVB-vehicle group was decreased compared with the normal group. The β-glucosidase activity of mice treated with putgyul extracts and retinoic acid was increased compared with the UVB-vehicle group (Figure 5B). β-glucosidase activity was increased in the presence of putgyul extracts in a dose-dependent manner, although not significantly. 

## 3. Discussion

As the average lifespan of humans increases, the effects of aging on the function and appearance of the skin are increasing. Skin aging is affected by a number of factors, including environmental stresses like UV light and mechanical stress [21]. Photoaged skin is characterized by wrinkles and uneven pigmentation [22,23]. The capability of a variety of natural topical agents to prevent aging has been studied [24,25]. In this study, we investigated the anti-photoaging effect of putgyul extracts from *Citrus unshiu* S.Marcov unripe citrus fruits. 

UVB results in oxidative stress that generates reactive oxygen species (ROS) in the skin. ROS oxidizes protein components of the cell membrane, which promotes aging [26,27]. Antioxidants are used to remove ROS. Presently, the antioxidative activity of putgyul extracts was measured by DPPH radical scavenging. Kamran Ghasemi et al. [28] measured the DPPH radical scavenging activity of various species of citrus fruit at ripening stage and found that it has excellent antioxidant ability. Yi et al. [29] reported that the vinegar produced by immature citrus fruit showed better antioxidant ability than the product produced by ripe citrus fruit, because the immature fruit had more polyphenol and flavonoid content than the ripe fruit. In our study, putgyul extract showed better antioxidant ability than the previously reported ripe citrus fruit. 

Skin tissue is composed of various types of collagen and ECM proteins. The structure of the tissue is maintained by the balance between the synthesis and collapse of extracellular components [30]. UV irradiation increases the activity of MMPs; the increased collagenase activity promotes collagen degradation and decreases synthesis [31]. The balance that is necessary for skin tissue integrity is disrupted and collagen fibers in the skin are damaged. Therefore, collagen is considered important in the prevention of photoaging [32]. Among several MMPs, MMP-1 plays a role in decomposing ECM dermal collagen that is mainly composed of type-1 collagen [33]. It is well known that natural plants have anti-photoaging effects through increasing collagen production by inhibition of MMP [34,35]. In this study, putgyul extracts decreased MMP-1 production in HDFs in a dose-dependent manner. In addition, the production of procollagen type-1 increased. These results indicate that putgyul extract suppresses ECM damage by inhibiting MMP-1.

Skin aging is associated with inflammatory reactions. UV irradiation upregulates the production of proinflammatory cytokines, such as IL-6, IL-1β, and TNF-α. UV also increases the expression of COX-2, which produces prostaglandin E_2_, and iNOS, which produces nitric oxide [11,36,37]. These inflammatory factors cause skin wrinkles. It has been reported that some natural substances have anti-photoaging effects via inhibition of inflammatory cytokines [10,17]. Similarly, putgyul extracts showed anti-photoaging effect by inhibiting these inflammatory cytokines. MMP-2 and 9 (gelatinase A and B, respectively) are activated by UV to break down ECM through the degradation of collagen, which promotes photoaging [38]. In this study, the expression of MMP-2 and 9 mRNA was inhibited in a concentration-dependent manner by putgyul extracts. However, only MMP-2 was significantly affected. When the expression of MMP-2 protein was measured by ELISA, the inhibitory effect of the putgyul extracts was also evident at the level of protein production. 

To investigate the effect of putgyul extracts on the prevention of photoaging in vivo, hairless mice were irradiated with UVB for 10 weeks. Skin elasticity associated with skin wrinkles is related to the tissue morphology of the epidermis. Photoaged skin has deep wrinkles [39,40] and displays increased epidermal thickness and collagen degradation due to increased production of MMPs [41]. Therefore, epidermal thickness and collagen change were observed through H&E staining and Masson’s trichrome staining. Putgyul extracts decreased skin wrinkle depth and collagen degradation by UVB irradiation. These results supported the inhibitory effect of putgyul extract on gene level of MMPs expression.

Skin also regulates the body’s homeostasis by reducing the TEWL [42]. However, TEWL is increased by UVB irradiation. Ceramide is a skin moisturizing indicators. Ceramide deficiency results in increased skin thickness and TEWL [43]. In this study, we evaluated the TEWL and activity of β-glucosidase, an enzyme involved in ceramide reorganization, in UVB-irradiated mice. UVB treatment resulted in increased TEWL, but putgyul extracts significantly reduced TEWL. In addition, putgyul extracts increased the activation of β-glucosidase enzyme in a concentration-dependent manner, but there was no significant difference among the experimental groups. These results support the view that putgyul extract has a skin protection effect against UVB irradiation damage.

The in vivo and in vitro experiments confirmed that putgyul extracts inhibit MMPs and inflammatory cytokines, and are effective for prevention of photoaging. In addition, skin wrinkle measurement, histological examination, TEWL determination, and β-glucosidase measurement support the idea that putgyul extracts protect skin from UVB irradiation damage. 

Further studies are needed to determine how putgyul extracts inhibit MMPs and inflammatory cytokines. The present study implicates putgyul extracts as a material capable of preventing photoaging of skin. 

## 4. Materials and Methods 

### 4.1. Materials

Dulbecco’s modified Eagle’s medium/Ham’s F-12 nutrient mixture (DMEM/F-12; 3:1 *v*/*v*), 1,1-diphenyl-2-picryl hydrazyl radical (DPPH), penicillin, streptomycin, retinoic acid, epigallocatechin gallate (EGCG), phenylmethylsulfonyl fluoride (PMSF), sodium taurocholate, and 4-methyllumbellifery-β-d-glucopyranoside were purchased from Sigma-Aldrich (St. Louis, MO, USA). Fetal bovine serum (FBS) and phosphate buffered saline (PBS) were purchased from Gibco (Gaithersburg, MD, USA). Dimethyl sulfoxide (DMSO) was obtained from Junsei Co. (Tokyo, Japan).

### 4.2. Sample Preparation

Unripe citrus of *Citrus unshiu* S.Marcov (putgyul) was collected in July 2016 from Jeju Island, Korea. The materials for extraction were cleaned, dried in a freeze dryer for 3 days and ground into a fine powder. The dried powder (50 g) was extracted with 50% ethanol at room temperature for 24 h and then evaporated under vacuum using a model N-3000 rotary evaporator (Eyela, Tokyo, Japan). 

### 4.3. Determination of Antioxidant Activity

DPPH radical-scavenging activity was determined as previously described [44]. Five concentrations of putgyul extracts were added to 100 µL of 0.4 mM DPPH. After mixing, they were reacted in the dark at room temperature for 10 min. The absorbance at 517 nm of each mixture was measured to evaluate the scavenging activity. Scavenging activity was expressed as the IC_50_ (the concentration required to remove 50% of the DPPH radical from the solution).

### 4.4. Cell Culture and Treatments

Human dermal fibroblasts (HDFs) isolated from human neonatal foreskin were purchased from the American Type Culture Collection (Manassas, VA, USA). HDFs were cultured on DMEM/F-12 containing 10% FBS, penicillin (100 IU/mL), and streptomycin (100 µg/mL) at 37 °C in a humidified atmosphere containing 5% CO_2_. Fibroblast cultures were subcultured by trypsinization and used between the fourth and tenth passages.

### 4.5. Cell Viability

Cell viability was determined using the MTT (3-[4,5-dimethylthiazol-2-yl]-2,5-diphenyl tetrazolium bromide) assay. The assay depends on the mitochondrial dehydrogenase activity in the viable cells to produce a dark blue formazan product. HDFs were cultured in DMEM/F-12 containing 10% FBS. Cells (5 × 10^4^) were dispensed in each well of a 96-well plate. After addition of samples to each well at different concentrations, the plates were incubated for 24 h at 37 °C in a CO_2_ incubator. After the incubation was completed and DMEM/F-12 was removed, 12 µL of 0.5% MTT and 100 µL of DMEM were added to the 96-well plate. The plates were then maintained in a CO_2_ incubator for 4 h to allow formazan formation to occur. The formazan was dissolved in 200 µL of DMSO and the absorbance at 570 nm was measured with a microplate reader (BIO-TEK Inc., Winooski, VT, USA). The results are presented as a percentage of control values.

### 4.6. Measurement of Intracellular Collagen

HDFs were cultured for 24 h in a 24-well plate with 5 × 10^4^ cells/well. After culturing, the samples were treated for 24 h in HDFs. After incubation for 24 h, the supernatant was collected and the amount of procollagen liberated into the medium was measured at 450 nm using procollagen type I C-peptide (PIP) ELISA Kit (Abcam, Cambridge, UK). The degree of collagen production was calibrated by total protein content, and the positive control group was compared using l-ascorbate. The amount of protein was determined by the Bradford method using bovine serum albumin (BSA) as a standard solution [39]. BSA was diluted in distilled water to 0.2, 5, 10 and 50 µg/mL. BSA solution was added to 1 mL of Bradford reagent in 20 µL of BSA solution for 5 min. Absorbance was measured at 595 nm. Using the same methods, the absorbance of the cell lysate of HDFs was measured and the amount of protein was calculated using a BSA standard curve.

### 4.7. Measurement of Intracellular Collagenase

HDFs were inoculated in wells of a 24-well plate (5 × 10^4^ cells/well) and cultured for 24 h. After culturing, the samples were treated for 48 h in HDFs. After 48 h of incubation, the activity of collagenase was measured at 450 nm using MMP-1 Human ELISA Kit (R&D System Inc., Minneapolis, MN, USA). MMP-1 activity was assessed by total protein content and compared with epigallocatechin gallate (EGCG) as a positive control. Protein quantification was performed using BSA as described above.

### 4.8. Animal Experiment

Seven-week old female HR-1 hairless mice were provided by Central Lab Animal Inc (Seoul, Korea). The Institutional Animal Care and Use Committee of Kangwon National University (No. KW-160919-1, 26 September 2016) approved all mouse experiments. The mice were divided into six groups, including a normal group (*n* = 5), UVB-vehicle group (*n* = 5), UVB-retinoic acid group (*n* = 5), UVB-putgyul 50 mg/kg group (*n* = 5), UVB-putgyul 100 mg/kg group (*n* = 5), and UVB-putgyul 200 mg/kg group (*n* = 5). The putgyul extracts and vehicle were orally administered for ten weeks. Body weight and food efficiency ratio were monitored every week. The minimal erythema dose (MED) on the skin of the mice was investigated. The mice were subjected to UVB radiation using a UVB lamp (Ieda Boeki, Tokyo, Japan). During the first week, 1 MED (100 mJ/cm^2^) of UVB was irradiated on the back of each mouse every day. From two to ten weeks, UVB irradiation was performed three times a week at 2 MED.

### 4.9. Wrinkle Measurement

The wrinkles were measured by modifying the previous study [45]. To investigate the wrinkle improvement of HR-1 skin induced by UVB irradiation, the mice were transiently anesthetized using ethyl ether, and the wrinkles were measured using a Dermobella wrinkle analyzer (Chowis, Seongnam, Korea) 4, 6, 8 and 10 weeks after UVB irradiation. Wrinkles were photographed at 400× magnification using a USB digital microscope.

### 4.10. Measurement of Transepidermal Water Loss (TEWL)

Mice were kept for 30 min in a room where the temperature and humidity were maintained (temperature 22 ± 1 °C, humidity 60 ± 5%). The back skin of each mouse was examined using Corneometer CM825 probe (Courage-Khazaka, Koln, Germany). The probe was placed in close contact with the surface of the back skin and lightly pressed to record the skin moisture content.

### 4.11. Measurement of β-Glucosidase Activity

To measure the activity of β-glucosidase (glucocereborsidase), the isolated epidermis was pulverized with PBS supplemented with 0.1 M phenylmethylsulfonyl fluoride and centrifuged at 10,000× *g* for 5 min at 4 °C. Fifty microliters of the supernatant was diluted with an equal volume of citrate-phosphate buffer (pH 5.6, 5 mM sodium taurocholate) containing 0.5 mM 4-methyllumbellifery-β-d-glucopyranoside (4-MUG). The solution was allowed to react for 60 min at 37 °C. The reaction was terminated by adding 1,250 µL of 200 mM carbonate-bicarbonate buffer (pH 10.5). The fluorescene intensity of 4-methyllumbelliferone (4-MU) produced by conversion of 4-MUG was measured with a model 300 spectrofluorimeter (Hitachi Co., Tokyo, Japan) at excitation and emission wavelengths of 360 and 450 nm, respectively. 4-MU concentrations ranging from 0 to 300 nM were used as the standard for fluorescence measurements. 

### 4.12. Real-Time Quantitative Reverse Transcription-Polymerase Chain Reaction (RT-PCR)

Quantitative real-time PCR was performed to study the anti-photoaging effects of putgyul extracts on cytokine gene expression from skin tissue. Total cellular RNA was extracted from skin tissue by the phenol-chloroform method (RNAzolB; Tel-Test Inc., Friendswood, TX, USA). Three micrograms of total RNA was used for cDNA synthesis that was carried out using the ReverTraAce-a-cDNA synthesis kit (Toyobo Co., Osaka, Japan). The 7500 Fast Real-Time PCR system (Applied Biosystems, Foster City, CA, USA) used for real-time quantitative PCR with the following primer sequences. COX-2, 5′-GGGTGTCCCTTCACTTCTTTCA-3′ and 5′-TGGGAGGCACTTGCATTGA-3′; IL-1β, 5′-CAACCAACAAGTGATATTCTCCATG-3′ and 5′-AGATCCACACTCTCAGCTGCA-3′; IL-6, 5′-TCCAGTTGCCTTCTTGGGAC-3′ and 5′-GTGTAATTAAGCCTCCGACTTG-3′; TNF-α, 5′-GGCTTTCCGAATTCACTGGAGCCT-3′ and 5′-CCCCGGCCTTCCAAATAAATACATTCATA-3′; iNOS, 5′-CGAAACGCTTCACTTCCAA-3′ and 5′-TGAGCCTATATTGCTGTGGCT-3′; MMP-2, 5′-CAGGGAATGAGTACTGGGTCTATT-3′ and 5′-ACTCCAGTTAAAGGCAGCATCTAC-3′; MMP-9, 5′-AATCTCTTCTAGAGACTGGGAAGGAG-3′ and 5′-AGCTGATTGACTAAAGTAGCTGGA-3′. 

The TaqMan probe containing carboxyfluorescein dye (Applied Biosystems) was used to demonstrate mRNA gene expression. Mouse glyceraldehyde-3-phosphate dehydrogenase probe set (4352339E, VIC/MGB Probe, Probe Limited; Applied Biosystems) was used as internal standard. The final concentration of the primer was 200 nM. 

The standard PCR conditions were 50 °C for 2 min, 94 °C for 10 min, then 40 cycles of 94 °C for 1 min and 60 °C for 1 min. The number of cycles in which the emission intensity of the sample rises above the baseline represents the relative quantity (RQ) and is proportional to the target concentration. Real-time PCR was analyzed according to the Applied Biosystems 7500 Fast Real-Time PCR system user manual. The relative quantitative value (RQ) of the target group was calculated by quantitative PCR.

### 4.13. Enzyme-Linked Immunosorbent Assay (ELISA)

Following the conclusion of the experiment, the expression level of MMP-2 protein in dorsal skin tissue extracted from each HR-1 mouse was measured using MMP-2 ELISA kit (R&D System Inc., Minneapolis, MN, USA). One hundred microliters of MMP-2 coated antibody was dispensed into each microwell and incubated at 4 °C for 16 h. Each well was washed with wash buffer prior to the addition of 200 µL of assay diluent and a 1-hour incubation at room temperature. After diluting the standard solution and diluting the supernatant 20 times, the microplate was washed and 100 µL of standard and supernatant was added to each well and incubated for 2 h at room temperature. The microplate was washed, 100 µL of working detector was added to each well, and the microplate was incubated for 1 h at room temperature. After another wash, 100 µL of substrate solution was added to each well prior to incubation in a dark room for 30 min at room temperature. Finally, 50 µL of stop solution was added to each well and the absorbance was measured at 450 nm on a microplate spectrophotometer.

### 4.14. Histological Analysis

Histological analysis was performed to determine epidermal thickness and collagen fiber analysis. Dorsal skin samples of experimental group were obtained after 10 weeks of UVB exposure, fixed with 10% formalin and embedded in paraffin. Sections were stained with hematoxylin and eosin (H&E) and Masson’s trichrome to examine epidermal thickness and stain collagen fibers, respectively. Epidermal thickness and collagen deposition were measured at 100× magnification using a digital microscope.

### 4.15. Statistical Analysis

Results are expressed as means ± standard deviation. The data were analyzed using ANOVA and Duncan’s multiple range tests. Significance was indicated at a *p-*value <0.05.

## 5. Conclusions

In this study, the oral administration of putgyul extracts prevented UV-induced skin aging. Putgyul extracts markedly suppressed MMPs, including MMP-1, MMP-2, and MMP-9, and inflammatory cytokines gene expression, including the expression of IL-1β, IL-6, COX-2, iNOS, and TNF-α. Putgyul extracts lessened skin wrinkle development, improved epidermal thickness, and lessened collagen degradation, TEWL, and the activity of β-glucosidase. Although the anti-photoaging mechanism still needs to be elucidated, the present results indicate the potential of putgyul extracts as a functional material in the prevention of skin damage caused by UVB.

## Abbreviations

UV	Ultraviolet
PIP	Procollagen type I c-peptide
MMP	Matrix metalloproteinase
RA	Retinoic acid
EGCG	Epigallocatechin gallate
